# Crystallisation Degree Analysis during Cryopreservation of Biological Tissue Applying Interval Arithmetic

**DOI:** 10.3390/ma16062186

**Published:** 2023-03-09

**Authors:** Alicja Piasecka-Belkhayat, Anna Skorupa

**Affiliations:** Department of Computational Mechanics and Engineering, Silesian University of Technology, Konarskiego 18A, 44-100 Gliwice, Poland

**Keywords:** crystallisation, vitrification, cryopreservation, directed interval arithmetic

## Abstract

This paper presents the numerical modelling of heat transfer and changes proceeding in the homogeneous sample, caused by the crystallisation phenomenon during cryopreservation by vitrification. Heat transfer was simulated in a microfluidic system in which the working fluid flowed in micro-channels. The analysed process included single-phase flow during warming, and two-phase flow during cooling. In the model under consideration, interval parameters were assumed. The base of the mathematical model is given by the Fourier equation, with a heat source including the degree of ice crystallisation. The formulated problem has been solved using the interval version of the finite difference method, with the rules of the directed interval arithmetic. The fourth order Runge–Kutta algorithm has been applied to determine the degree of crystallisation. In the final part of this paper, examples of numerical computations are presented.

## 1. Introduction

Cryopreservation is the process of slowing down or stopping the biological activity of tissues or cells by lowering the temperature below zero (in °C), and then recovering that activity after returning to the physiological temperature. The main purpose of cryopreservation is to preserve tissues and cells without losing their essential characteristics, such as vital functions or mechanical properties. Cryopreservation is used, among other things, to preserve organs for transplantation and during stem cell research [1,2,3].

When considering heat transfer during cryopreservation, it is important to take into account the physical phenomena occurring in biological structures. One of these is the process of crystallisation. Crystallisation is a phenomenon involving the formation of a crystalline phase. In this phase, molecules or atoms are strongly organised into a lattice called a crystal. The course of crystallisation depends on several factors, such as the homogeneity of the liquid, the pressure or the cooling rate [4,5].

The two main stages of crystallisation are nucleation and ice crystal growth. Nucleation is the beginning of a phase transition over a small area, during which a crystalline phase is formed. Nucleation is the result of local fluctuations occurring in a metastable homogeneous phase (e.g., in an overcooled liquid) on a molecular scale. In the case of ice crystal growth, soon after the formation of the first seed crystal, a nucleus, which is a point of convergence for neighbouring molecules, is formed. When the solution is metastable due to supercooling or supersaturation, the crystal develops successive layers around it, thus increasing its dimensions. The growth rate, which is a constant parameter specific to the process, can then be determined [4,5,6].

During cryopreservation, these phenomena occur in both intracellular and extracellular areas when the temperature decreases below the freezing point. The process of water crystallisation in tissues and cells can lead to irreversible damage. To prevent this, the cryoprotectant (CPA) concentration and cooling rate are “controlled” [2,3].

One method of cryopreservation is vitrification. Cryopreservation by vitrification uses a high concentration of CPA and rapid cooling, in which the transition from the liquid to the vitreous state takes place. Vitrification is considered a very effective method for cryopreservation of cells and tissues. Vitrification has great advantages over the traditional slow freezing method [7,8]. The vitrification process is easy to perform, as the cooling rate does not need to be controlled. A major advantage of vitrification is that it avoids the formation of intracellular ice [1,9,10].

In general, the opportunity to develop the cryopreservation process comprises mathematical modelling and undertaking computer simulations. For this purpose, various numerical methods are used, such as the finite different method (FDM), the finite element method (FEM), the finite volume method (FVM), or the finite integration technique (FIT) [11]. A numerical approach can be used to investigate the behaviour of different biological tissues, such as articular cartilage [12,13,14,15,16] and stem cells [17,18,19], during cryopreservation.

In the process of cryopreservation, the phenomenon of heat transfer is primarily considered. The fundamental relationship describing thermal phenomena is depicted by the Fourier equation [20]. Other formulas applied to the bioheat transfer issue are the Pennes equation [21], the Cattaneo–Vernotte equation [22,23,24], and the dual phase lag (DPL) model. Bioheat transfer modelling describes phenomena that occur not only during cryopreservation by slow freezing [17] and vitrification [12,15,25], but also other examples of thermal process modelling applications, such as the characterisation of cryosurgery [26,27,28,29,30] or the destruction of biological tissue under the influence of a magnetic field [11,31].

The phenomenon of cryopreservation can be described by a mathematical model.

It is based on an equation defining the heat transport, such as the equation proposed by Fourier [20]. When thermal transfer is analysed, it is also necessary to consider the coupled crystallisation process [7,8,25,32].

The crucial components of the energy equation are the thermophysical parameters. The parameters defining the tissues and other biological structures are not inherently characterised in a deterministic manner. These quantities are generally determined during experimental studies, so there is a certain element of randomness. Despite this, scientists often take averages of measured quantities and then study a deterministic model. The second approach to modelling is to use stochastic models, for which a lot of computational time is usually spent.

The main idea of this paper was to use a different approach to modelling the vitrification process, namely interval numbers, which were used to determine some parameters such as thermal conductivity and specific heat. The basis of the mathematical model of the task defined in this way is the interval Fourier equation, with an interval source function describing the crystallisation process. During the numerical simulation, the implicit scheme of the interval finite difference method was used. In addition, the fourth-order Runge–Kutta algorithm was used to linearise the interval source component, taking into account the degree of ice crystallisation. All numerical calculations were carried out according to the rules of directed interval arithmetic. As a result, the computational results were obtained in the form of intervals [33,34,35,36,37,38].

Another aim of this study was to compare the results of a numerical simulation considering interval parameters with the data presented in [8]. In this paper, a new type of device is described to significantly increase the cooling and warming rates in the vitrification process. The experiment uses a microfluidic system, in which microchannels are used to supply cooling and warming media and to increase heat transfer to the sample.

## 2. Materials and Methods

In this research, a microfluidic system with microchannels is modelled. The design is created according to the geometry proposed by Tuckerman and Pease [39] and Zhou et al. [8]. The central part of the system consists of microcavities, in which a thin layer of solution with a biological sample is placed. The relatively small thickness of the sample achieved by the microfluidic construction promotes a regular temperature distribution inside. This layer is surrounded by a casing in which microchannels are extruded. Placed in this element is the working fluid that regulates the rate of cooling.

Depending on the phase of the process, liquid nitrogen and water are used during cooling and warming, respectively. It is also worth mentioning that the casing of the entire system is made of silicone. The sample layer for the simulation is replaced by a cell-free solution, which consists of 55% water and 45% ethylene glycol (EG) [8].

**Figure 1:**
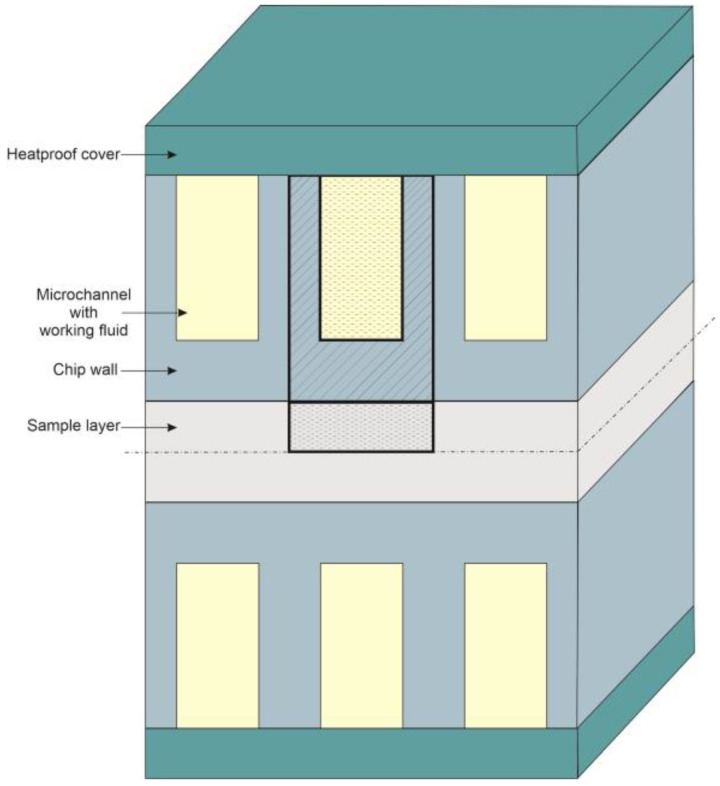
Schematic model of microfluidic system.

Figure 1 shows a schematic presentation of the modelled system. A single cell of the entire device, where thermal transitions are studied, is highlighted [8].

### 2.1. Governing Equations

**Figure 2:**
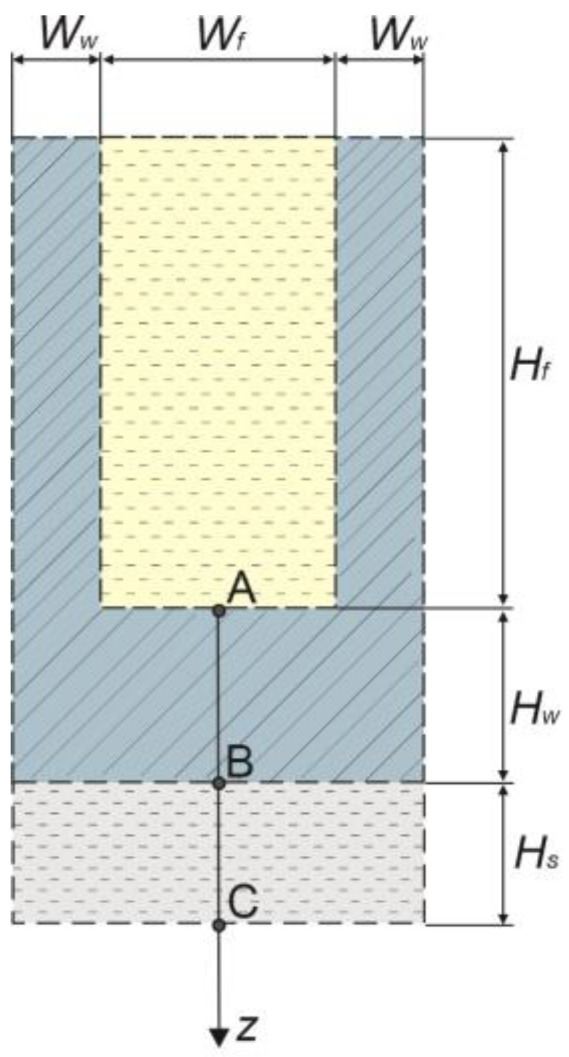
One-dimensional model of the microfluidic system.

Considering that the cell suspension is kept as a thin layer in the chip, the heat transfer from the sample to the outside can be treated as a one-dimensional task. Referring to the system shown in Figure 1, the one-dimensional problem can be visualised as illustrated in Figure 2 [8]. The marked points A, B, and C are the boundary nodes on which the boundary conditions are applied. The relationships at the boundary nodes will be described in more detail later in this work.

When the temperature of the liquid approaches its freezing point, solidification of the liquid begins. Three models can be used to describe the solidification process: the uncoupled method, the Stefan approach and the zone model. In the uncoupled method, latent heat is neglected during freezing, meaning that the degree of ice crystallisation does not affect internal heat sources. The Stefan problem assumes a moving boundary between the solid and the liquid, additionally taking into account the transition zone between these areas. This paper adopts a zone model in which the crystallisation is simulated as a propagation zone, which can cover the whole area or be very narrow. The size of the zone depends primarily on the material parameters and the process conditions. The zone model assumes that the total heat, which consists of latent heat and sensible heat, can be determined from the enthalpy function [2,8,40,41].

The energy equation governing the temperature distribution inside the microfluidic chip for the one-dimensional problem can be defined as the interval Fourier equation [20]:

(1) $$\frac{\partial \left(\bar{c}\left(\bar{T}\right)\rho \bar{T}\left(z,t\right)\right)}{\partial t}=\frac{\partial }{\partial_{z}}\left(\bar{\lambda }\left(\bar{T}\right)\frac{\partial \bar{T}\left(z,t\right)}{\partial_{z}}\right)+{\bar{S}}_{h},$$

where $\bar{T}$ is the interval temperature, $\bar{\lambda }$ is the interval thermal conductivity, $\bar{c}$ is the interval specific heat, ρ is the density, while ${\bar{S}}_{h}$ is the interval heat source term.

For the chip wall, ${\bar{S}}_{h}$ is equal to zero because there is no heat source there. For the sample layer, on the other hand, the heat source term is expressed as [7,25,41]:

(2) $${\bar{S}}_{h}=\rho_{h}L_{h}\frac{\partial \bar{\chi }}{\partial t},$$

where $\bar{\chi }$ represents the interval degree of ice crystallisation ($\bar{0}<\bar{\chi }<\bar{1}$), $\rho_{h}$ is the density of water, while $L_{h}$ is the latent heat of water. Note that instead of the thermophysical parameters of the biological sample, values for water are implemented. This assumption is introduced because the thermal properties of biological cells are similar to those of water, which is their main construction component [25].

The growth rate of $\bar{\chi }$ (denoted by ${\bar{\chi }}^{\prime }=\partial \bar{\chi }/\partial t$), which describes the crystallisation process, is determined using the following non-isothermal kinetic equation proposed by Boutron and Mehl [42]:

(3) $$\frac{\partial \;\bar{\chi }}{\partial t}={\bar{\chi }}^{\prime }(\bar{\chi },\;T)=k_{a}{\bar{\chi }}^{\frac{2}{3}}\left(1-\bar{\chi }\right)\left(T_{m}-\bar{T}\right)e^{-\frac{Q}{R\bar{T}}},$$

where $k_{a}$ is the characteristic coefficient depending on the solution composition, $T_{m}$ is the freezing (melting) temperature, *Q* is the activation energy, and *R* is the gas constant (*R* = 8.314 J·mol^−1^·K^−1^).

All chemical reactions, including exothermic reactions such as crystallisation, need activation energy to start. The activation energy is related to the nucleation and growth process. Values for the activation energy of crystallisation *Q* are determined by various methods, such as the Kissinger method and its modifications, the Augis–Bennett method, the Baswell method, and the Gao–Wang method. These methods study the dependence of the heating (cooling) rate on the crystallisation temperature [43]. Looking at Equation (3), it can be seen that the higher the ratio of the activation energy *Q* to the average kinetic energy $\left(R\bar{T}\right)$, the lower the growth rate of $\bar{\chi }$.

Boundary conditions have to be attached to the mathematical model. The entire structure is surrounded by thermal insulation (see Figure 1—heatproof cover); hence the heat flux between the devices and the environment is neglected.

The main source of thermal changes in the system is the working fluid contained in the microchannels. As mentioned above, during cooling and warming, liquid nitrogen and water are used. In the system shown in Figure 2, point A is the node of contact between the model and the working fluid. The heat flux at point A is described by a boundary condition of the third type. This condition was extended to include the geometry of the microchannel. The relation has the following form [8]:

(4) $$\bar{q}(z,t)\cdot \left(W_{f}+2W_{w}\right)=\alpha \left(\bar{T}(z,t)-T_{f}\right)\left(W_{f}+2\bar{\eta }H_{f}\right),$$

where $\bar{q}$ is the interval heat flux, *W_f_*, *W_w_*, *H_f_* are the microchannel dimensions (see Figure 2), *T_f_* is the temperature of the working fluid, α is the external heat transfer coefficient, and $\bar{\eta }$ is the interval fin efficiency. The interval heat flux is defined as [40]:

(5) $$\bar{q}(z,t)=-n\bar{\lambda }\frac{\partial \bar{T}(z,t)}{\partial z},$$

where **n** is the normal vector.

The interval fin efficiency is calculated from the relation [8]:

(6) $$\bar{\eta }=\frac{\tanh\left(\bar{m}H_{f}\right)}{\bar{m}H_{f}},$$

where $\bar{m}$ is the interval fin parameter given by [8]:

(7) $$\bar{m}=\sqrt{\frac{2\alpha }{{\bar{\lambda }}_{w}(\bar{T})\cdot 2W_{w}}},$$

where ${\bar{\lambda }}_{w}$ is the interval thermal conductivity for the chip wall.

Furthermore, the external heat transfer coefficient is also worth noting as a component of Equation (3). In fact, it is difficult to determine its value experimentally for a working fluid in a microchannel. Therefore, a theoretical relationship has been developed to calculate it. In addition, two types of flow for microchannels are considered: single-phase flow and two-phase flow. During cooling, liquid nitrogen is the working fluid, and as a result, in the microchannel both liquid nitrogen and small quantities of vapour nitrogen exist; therefore, a two-phase flow should be modelled in this case. On the other hand, for warming, water is applied. Water as the working fluid is only present in liquid form. Hence, it is a single-phase flow. A discussion about the external heat transfer coefficient is provided, e.g., in [8,39,44]. Our work assumes constant values for the external heat transfer coefficient for individual flows.

Let us also introduce the other boundary conditions. At point B is the contact between the chip wall and the sample layer. A boundary condition of the fourth type is implemented at this node [40]:

(8) $$\{\begin{array}{c} -n{\bar{\lambda }}_{w}\frac{\partial {\bar{T}}_{w}(z,t)}{\partial z}=-n{\bar{\lambda }}_{f}\frac{\partial {\bar{T}}_{f}(z,t)}{\partial z}\\ {\bar{T}}_{w}\left(z,t\right)={\bar{T}}_{f}\left(z,t\right) \end{array},$$

where subscripts *w* and *s* denote the chip wall and the sample layer domain, respectively. Note that it is assumed that the contact is ideal.

The whole system is symmetrical, therefore, an adiabatic condition is defined at point C [8,40]:

(9) $$\bar{q}(z,t)=-n\bar{\lambda }\frac{\partial \bar{T}(z,t)}{\partial z}=\bar{0}.$$

In addition, the initial conditions complete the mathematical model. It is necessary to determine the temperature distribution and the degree of crystallisation in the sample domain at time *t* = 0 [8]:

(10) $$\left\{ \begin{array}{l} \bar{T}(z,0)=T_{0}\\ \bar{\chi }(z,0)=\chi_{0} \end{array}\right.,$$

where $T_{0}$ and $\chi_{0}$ are the initial values of the temperature and the degree of crystallisation, respectively.

### 2.2. Numerical Model

The interval finite difference method was used to solve an unsteady heat transfer problem (see Equation (1)). The finite difference method is a very good tool for solving non-linear equations. The non-linearity of Equation (1) is related to the varying values of the thermophysical parameters $\bar{c}\left(\bar{T}\right)$ and $\bar{\lambda }\left(\bar{T}\right)$, as well as the interval heat source term ${\bar{S}}_{h}\left(\bar{T}\right)$. The numerical model of thermal processes proceeding in the microfluidic chip is based on the finite difference method in the version presented in [40,45].

It should be remembered that calculations are performed using the rules of interval arithmetic described briefly, e.g., in [33,38].

**Figure 3:**
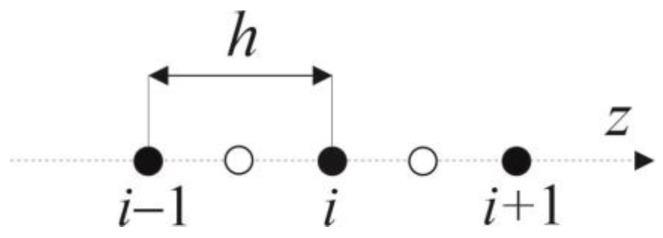
Three-points star.

Firstly, a time grid with a constant step Δ*t* and a geometrical mesh with a constant step *h* are introduced. Figure 3 presents the concept of a three-points star, which is applied to create the geometrical mesh.

Assuming weak non-linearity of the specific heat, the differential equation for the internal nodes *i*, corresponding to the interval Fourier equation written in the implicit scheme, is of the form [45]:

(11) $${\bar{c}}_{i}^{f-1}\;\rho_{i}\frac{{\bar{T}}_{i}^{f}-{\bar{T}}_{i}^{f-1}}{\Delta t}=\frac{2}{h^{2}}\frac{{\bar{\lambda }}_{i-1}^{f-1}\;{\bar{\lambda }}_{i}^{f-1}}{{\bar{\lambda }}_{i-1}^{f-1}+{\bar{\lambda }}_{i}^{f-1}}\left({\bar{T}}_{i-1}^{f}-{\bar{T}}_{i}^{f}\right)+\frac{2}{h^{2}}\frac{{\bar{\lambda }}_{i+1}^{f-1}\;{\bar{\lambda }}_{i}^{f-1}}{{\bar{\lambda }}_{i+1}^{f-1}+{\bar{\lambda }}_{i}^{f-1}}\left({\bar{T}}_{i+1}^{f}-{\bar{T}}_{i}^{f}\right)+{\left({\bar{S}}_{h}\right)}_{i}^{f},$$

where *f* means a moment of time, the time step $\Delta t=t^{f}-t^{f-1}$ and

(12) $${\bar{c}}_{i}^{f-1}=\bar{c}\left({\bar{T}}_{i}^{f-1}\right),$$

while

(13) $${\bar{\lambda }}_{k}^{f-1}=\bar{\lambda }\left({\bar{T}}_{k}^{f-1}\right),$$

where *k* denotes the node number (*k* = *i* − 1, *i*, *i* + 1).

Equation (11) can be written in the following form [45]:

(14) $$A_{i}{\bar{T}}_{i-1}^{f}+B_{i}{\bar{T}}_{i}^{f}+C_{i}{\bar{T}}_{i+1}^{f}={\bar{T}}_{i}^{f-1}+\frac{\Delta t}{{\bar{c}}_{i}^{f-1}\;\rho_{i}}{\left({\bar{S}}_{h}\right)}_{i}^{f},$$

where

(15) $$\begin{array}{c} A_{i}=-\frac{2\Delta t}{h^{2}{\bar{c}}_{i}^{f-1}\;\rho_{i}}\cdot \frac{{\bar{\lambda }}_{i-1}^{f-1}\;{\bar{\lambda }}_{i}^{f-1}}{{\bar{\lambda }}_{i-1}^{f-1}+{\bar{\lambda }}_{i}^{f-1}},\;\;\;\;\;\;B_{i}=\frac{2\Delta t}{h^{2}{\bar{c}}_{i}^{f-1}\;\rho_{i}}\cdot \left(\frac{{\bar{\lambda }}_{i-1}^{f-1}\;{\bar{\lambda }}_{i}^{f-1}}{{\bar{\lambda }}_{i-1}^{f-1}+{\bar{\lambda }}_{i}^{f-1}}+\frac{{\bar{\lambda }}_{i+1}^{f-1}\;{\bar{\lambda }}_{i}^{f-1}}{{\bar{\lambda }}_{i+1}^{f-1}+{\bar{\lambda }}_{i}^{f-1}}\right),\\ C_{i}=-\frac{2\Delta t}{h^{2}{\bar{c}}_{i}^{f-1}\;\rho_{i}}\cdot \frac{{\bar{\lambda }}_{i+1}^{f-1}\;{\bar{\lambda }}_{i}^{f-1}}{{\bar{\lambda }}_{i+1}^{f-1}+{\bar{\lambda }}_{i}^{f-1}}. \end{array}$$

The resulting system of interval equations supplemented with boundary conditions (see Equations (4) and (9)) can be solved using the Thomas method. It should be noted that the implicit scheme is always stable, and there are no restrictions on the allowable time step values [40,45].

In the model presented, the interval source component (see Equation (2)) must also be linearised [8]:

(16) $${\bar{S}}_{h}\left({\bar{\chi }}_{i}^{f}\right)=\rho_{h}L_{h}\frac{{\left(\bar{\chi }\right)}_{i}^{f}-{\left(\bar{\chi }\right)}_{i}^{f-1}}{\Delta t}.$$

Because of the difficulty of calculating the analytical expression $\bar{\chi }\left(\bar{T}\right)$ appearing in the non-isothermal kinetic equation (see Equation (3)), in this paper the values of the interval degree of ice crystallisation $\bar{\chi }$ and its growth rate ${\bar{\chi }}^{\prime }$ have been calculated numerically using the fourth-order Runge–Kutta algorithm according to the following formulas [8]:

(17) $${\bar{\chi }}^{f+1}={\bar{\chi }}^{f}+{\left({\bar{\chi }}^{\prime }\right)}^{f+1}\cdot \Delta t,$$

where

(18) $${\left({\bar{\chi }}^{\prime }\right)}^{f+1}=\frac{{\left({\bar{\chi }}^{\prime }\right)}_{1}+2{\left({\bar{\chi }}^{\prime }\right)}_{2}+2{\left({\bar{\chi }}^{\prime }\right)}_{3}+{\left({\bar{\chi }}^{\prime }\right)}_{4}}{6}$$

for

(19) $$\left\{ \begin{array}{l} {\left({\bar{\chi }}^{\prime }\right)}_{1}={\bar{\chi }}^{\prime }({\bar{\chi }}^{f},\;{\bar{T}}^{f})\\ {\left({\bar{\chi }}^{\prime }\right)}_{2}={\bar{\chi }}^{\prime }\left({\bar{\chi }}^{f}+\frac{{\left({\bar{\chi }}^{\prime }\right)}_{1}}{2}\Delta t,\;{\bar{T}}^{f}+\frac{\Delta {\bar{T}}^{f}}{2}\right)\\ {\left({\bar{\chi }}^{\prime }\right)}_{3}={\bar{\chi }}^{\prime }\left({\bar{\chi }}^{f}+\frac{{\left({\bar{\chi }}^{\prime }\right)}_{2}}{2}\Delta t,\;{\bar{T}}^{f}+\frac{\Delta {\bar{T}}^{f}}{2}\right)\\ {\left({\bar{\chi }}^{\prime }\right)}_{4}={\bar{\chi }}^{\prime }\left({\bar{\chi }}^{f}+{\left({\bar{\chi }}^{\prime }\right)}_{3}\Delta t,\;{\bar{T}}^{f}+\Delta {\bar{T}}^{f}\right) \end{array}\right.,$$

where $\Delta {\bar{T}}^{f}={\bar{T}}^{f+1}-{\bar{T}}^{f}$.

The numerical simulation assumed a temperature’s dependence on thermal conductivity and the specific heat of silicon. Measurement points given in the literature [46,47,48] in the temperature range 20–300 K (from −253 °C to 27 °C) were approximated by a fifth- and fourth-degree polynomial, respectively, using the linear regression method (*R*^2^ = 0.989 for the thermal conductivity and *R*^2^ = 0.999 for the specific heat):

(20) $$\begin{array}{c} \begin{array}{cc} {\bar{\lambda }}_{w}(\bar{T})= & 1.3496\cdot 10^{-8}{\bar{T}}^{5}+1.1636\cdot 10^{-5}{\bar{T}}^{4}+0.0024\;{\bar{T}}^{3}+0.1416\;{\bar{T}}^{2}\\ & -2.0261\;\bar{T}+54.3813, \end{array}\\ \begin{array}{cc} {\bar{c}}_{w}(\bar{T})= & 2.4923\cdot 10^{-7}{\bar{T}}^{4}+9.1657\cdot 10^{-5}{\bar{T}}^{3}\;+0.0023{\bar{T}}^{2}+1.395\bar{T}\\ & +\;677.6804. \end{array} \end{array}$$

The thermal conductivity and the specific heat of the EG solution were also expressed as temperature-dependent polynomial functions using a linear regression method. These relationships have been derived from the literature, which presents the functions proposed directly by the producer of the EG solutions [8,49].

(21) $$\begin{array}{l} {\bar{\lambda }}_{s}(\bar{T})=\left(-2.7041\cdot 10^{-2}{\bar{T}}^{2}-17.741\;\bar{T}+1442.8\right)/1000,\\ {\bar{c}}_{s}(\bar{T})=2.8467\;\bar{T}+2727.7. \end{array}$$

Note that the interval temperature in Equations (18) and (19) should be given in °C.

## 3. Results

As mentioned, the study modelled a one-dimensional system that analyses heat transfer in a single unit cell of the whole structure (see Figure 2). The dimensions of a single unit cell, parameters of the working fluid, crystallisation properties of the sample layer (composition of water and EG), and others are presented in Table 1.

The time and geometric grid parameters are specified for the model: Δ*t* = 0.01 s, *h* = 2.0202 × 10^−6^ m, where the number of nodes is *m* = 100 (the number of elements is *l* = 99). The initial parameters are as follows: *T*_0_ = 22 °C and χ_0_ = 0. The calculations have been performed using the finite difference method supplemented by interval arithmetic rules.

**Figure 4:**
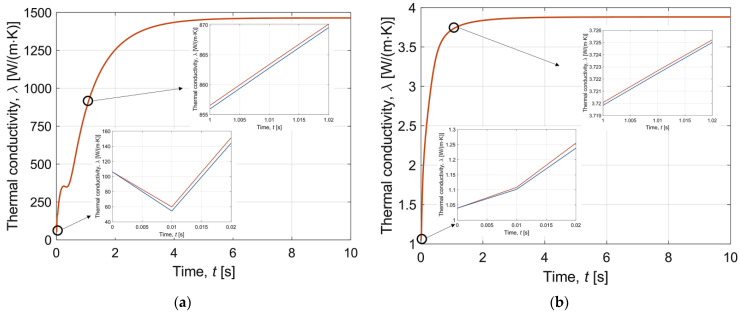
Interval thermal conductivity as a function of time during cooling for (**a**) chip wall; (**b**) sample layer.

**Figure 5:**
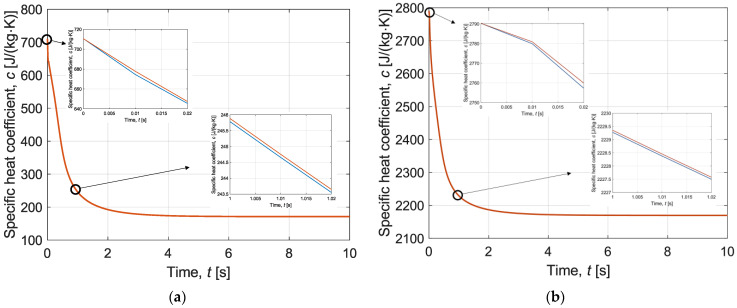
Specific heat coefficient as a function of time during cooling for (**a**) chip wall; (**b**) sample layer.

Firstly, Figure 4 and Figure 5 present the interval thermal conductivity and the interval specific heat as a function of time, respectively. Diagrams with enlarged sections have been prepared for both the chip wall made of silicone and the sample layer (water and EG solution). These curves clearly indicate that the thermophysical parameters are temperature-dependent and change during the simulation. Therefore, it is reasonable to introduce them in the form of a function. In addition, it is the thermal conductivity and the specific heat for both the chip wall and the sample layer that are the interval numbers in the model. The deviation from the value estimated from Equations (18) and (19) is equal to 5% ($\bar{\lambda }\left(\bar{T}\right)=\left[\lambda -0.05\lambda ;\;\lambda +0.05\lambda \right]$ and $\bar{c}\left(\bar{T}\right)=\left[c-0.05c;c+0.05c\right]$).

**Figure 6:**
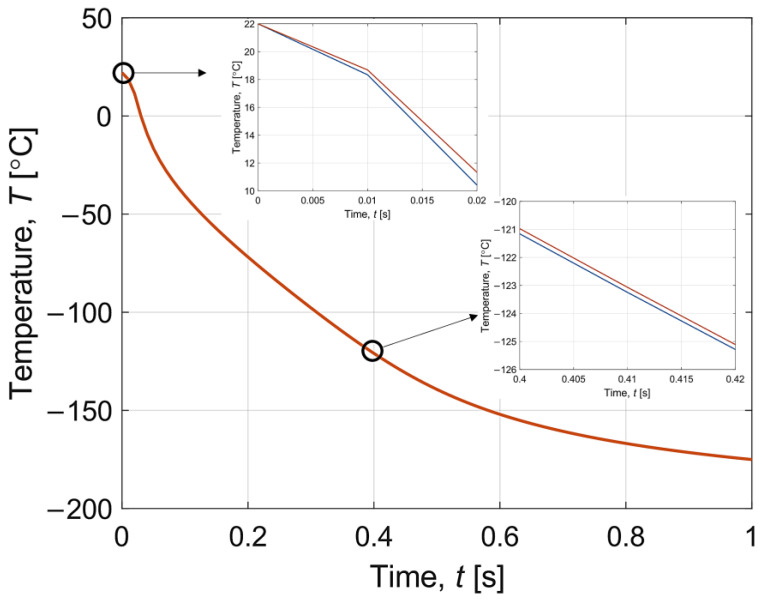
History of interval temperature during cooling in time 0–1 s.

The results in Figure 6 demonstrate the interval temperature changes during cooling at a point located in the central part of the sample layer (*z* = *H_w_* + *H_s_*). The width of the interval is relatively small; hence, an approximation is prepared for 0–0.02 s and 0.40–0.43 s, which confirms that the resulting temperature course is in the form of intervals. Note that the red line and the blue line represent the upper and lower limits of the interval, respectively. It can be seen that stabilisation of the temperature to the assumed temperature by working fluid is achieved within a few seconds. In the whole sample, the minimum temperature is reached after 14.1 s.

**Figure 7:**
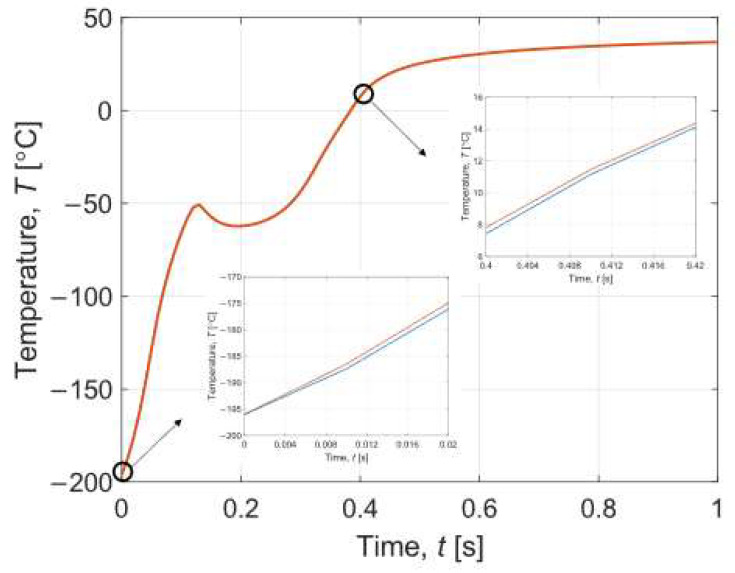
History of interval temperature during warming in time 0–1 s.

Figure 7 also illustrates the interval temperature variations over time in the central part of the sample layer for the warming process. For this plot, certain sections have also been zoomed in to show the intervals. The temperature of the entire sample is 40 °C after a warming time equal to 7.15 s. It can be observed that there is a decrease in temperature at a certain moment. It can be related to the recrystallisation phenomenon that occurs at this time.

**Figure 8:**
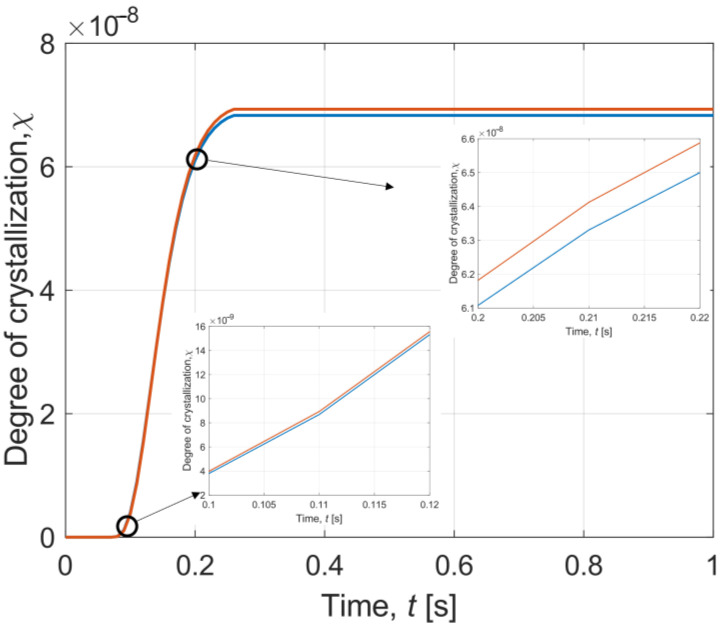
History of interval degree of crystallisation during cooling in time 0–1 s.

Afterwards, results describing crystallisation phenomena have been prepared. Figure 8 depicts modifications of the interval degree of crystallisation during cooling in time 0–1 s in the central part of the sample layer, with enlarged fragments for 0.1–0.12 s and 0.2–0.22 s. It can be noted that the crystallisation process stabilises after some time.

**Figure 9:**
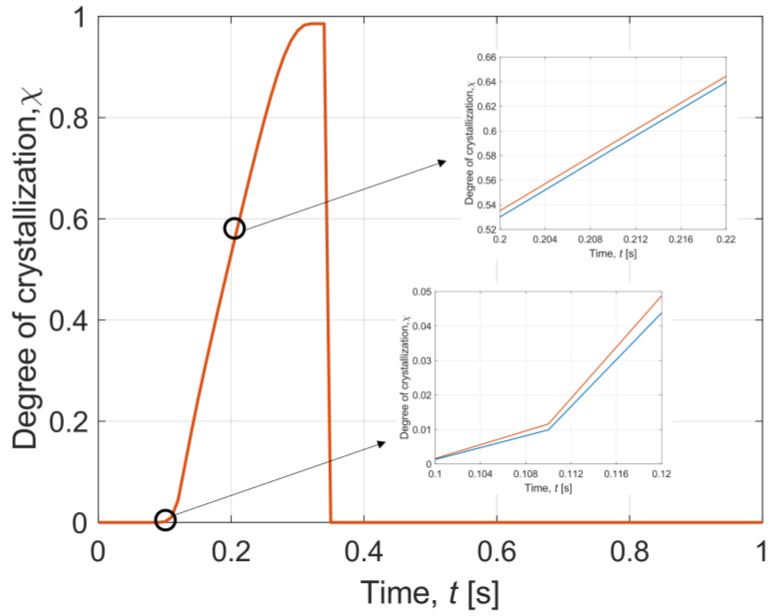
History of interval degree of crystallisation during warming in time 0–1 s.

Figure 9 presents the interval degree of crystallisation during warming in time 0–1 s in the central part of the sample layer. During warming, a sharp peak of the interval degree of crystallisation can be observed. It can be stated that this occurs when the temperature distribution in the sample is between −90 °C and −20 °C. As one reads, it is a dangerous temperature region (DTR) [8,51]. This is the critical moment when ice crystallisation occurs. Therefore, it is important to minimise the duration of the DTR during cryopreservation. In our instance, DTR passing time is equal to 0.2 s for cooling and 0.26 s for warming.

During cooling, the highest average value of the degree of crystallisation is χ = 1.075 × 10^−7^ while for warming it is χ = 0.999. It can be said that the lower risk of cell damage caused by crystallisation is during the cooling process. However, for warming, the degree of crystallisation increases as a result of recrystallisation and then decreases to 0 after exceeding the DTR; while for cooling, the degree of crystallization remains constant after passing DTR.

The results obtained are also compared with the data reported by Zhou et al. [8]. It can be said that the course of the temperature and the degree of crystallisation are similar to our plots. In Zhou et al. [8], equally rapid cooling and warming occur, as shown in Figure 6 and Figure 7 for our case. In contrast, the temperature decrease during warming in Zhou et al. [8] is not so visible compared to our $\bar{T}$(*t*) curve in Figure 7. This indicates a smaller effect of recrystallisation than in our simulation. On the other hand, analysing the graphs of the degree of crystallisation, it can be seen that the characteristics of the results are the same. The degree of crystallisation in Zhou et al. [8] during cooling maintains a constant value after reaching a certain limit, while during warming there occurs a sudden peak caused by recrystallisation, which tends to zero after exceeding DTR. One can observe a significant difference of several orders of magnitude between the results presented in our paper and those of Zhou et al. [8].

It is also worth noting other crucial values obtained by Zhou et al. [8]. During cooling, the transition time through the DTR is equal to 0.042 s and the maximum crystallisation rate is less than 2 × 10^−11^. Whereas for heating, the passing through the DTR lasts 0.057 s and the crystallisation rate is less than 2.4 × 10^−3^. These results are significantly lower than indicated in our simulation. However, it is important to consider a criterion that defines a certain maximum value of the degree of crystallisation, above which crystallisation can damage the sample. It is assumed that χ < 10^−6^, thus, both the results from Zhou et al. [8] and from our model fulfill this criterion for cooling and heating after recrystallization disappears.

Note that Figure 4, Figure 5, Figure 6, Figure 7, Figure 8 and Figure 9 contain the results as interval numbers. The width of the intervals is small, and it is often difficult to indicate this. Therefore, in Table 2, there are example results for a given point in the central part of the sample layer (*z* = *H_w_* + *H_s_*) at selected moments of the simulation time.

**Figure 10:**
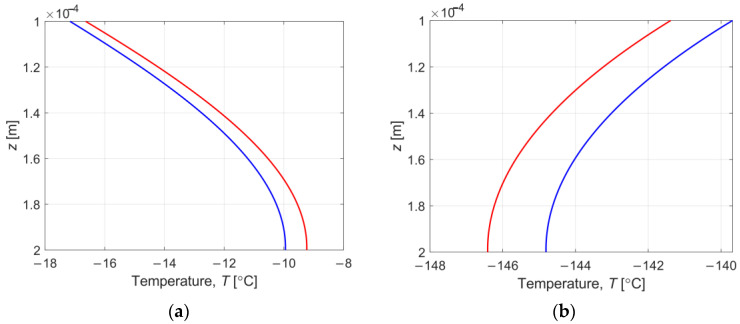
Interval temperature in the cross-section of the sample at simulation time *t* = 0.04 s: (**a**) for cooling; (**b**) for warming.

Finally, Figure 10 shows the changing of the interval temperature for cooling (Figure 10a) and for warming (Figure 10b) in the cross-section of the sample at simulation time *t* = 0.04 s. It can be clearly observed that the point of contact between the sample and the chip wall reacts most rapidly to the temperature change caused by the working fluid interacting with the casing. Indeed, after a certain period of time, the interval temperature and the interval degree of crystallisation across the entire domain change.

## 4. Discussion

The paper describes the mathematical and numerical model of a microfluidic system used to perform cryopreservation by vitrification. The device was modelled based on the concepts proposed by Tuckerman and Pease [39] and Zhou et al. [8]. This construction improves the cooling rate during vitrification.

Thermal processes and crystallisation phenomena defined by the Fourier equation are coupled to the non-isothermal kinetic equation developed by Boutron and Mehl. The simulation was carried out using FDM and the fourth-order Runge–Kutta algorithm.

In addition, our case considers the non-linear nature of the thermophysical parameters. The introduction of interval numbers allows us to interpret the results closer to reality compared to a deterministic approach.

The interval results obtained were compared with simulations performed by Zhou et al. [8]. It can be observed that the temperature variations in the central part of the sample coincide with each other. Thermal stabilisation across the entire cross-section of the sample occurs relatively quickly, confirming the main postulate of vitrification about the high cooling rate. Analysing the interval temperature distribution in the cross-section of the sample layer, it can be observed that all changes occur most rapidly in the region of contact between the cell-free solution with the casing (point B).

In this process, it is important that the time is kept short when the sample is in the DTR region. This reduces the risk of ice crystals formation, which causes damage to the biological structures. It can be said that the duration of the DTR in our case is relatively short compared to the whole process. Unfortunately, the DTR values presented by Zhou et al. are lower than in our case, which may suggest differences in the model used [8].

Another marker of destruction in a biological sample is the degree of crystallisation. According to the literature [8,42,51], the degree of crystallisation should be less than 10^−6^. It can be observed that for cooling, this condition is achieved and the phenomenon of ice crystallisation can be neglected. For warming, the degree of crystallisation is higher than the given maximum value. However, after the recrystallisation time, the rate rapidly falls to zero after passing through the DTR as a result of heating the sample structure. Thus, it can be assumed that the crystallisation is not damaging the sample.

## 5. Conclusions

The analysis of the effect of CPA on the sample as a result of osmotic transport was omitted from the study. Therefore, it seems reasonable to carry out future research on this problem. This will involve introducing the actual biological structure into the model. This approach will make it possible to investigate sample damage as a result of cytotoxicity and osmotic shock.

## Figures and Tables

**Table 1:** Input data of the model.

Parameter		Value
Dimensions [8,39]		
*W_f_*	[m]	5 × 10^−5^
*W_w_*	[m]	2.5 × 10^−5^
*H_f_*	[m]	3.5 × 10^−4^
*H_w_*	[m]	1 × 10^−4^
*H_s_*	[m]	1 × 10^−4^
Working fluid parameters [8,44,50]		
*T_f_*	[°C]	−196 (for cooling)/40 (for warming)
α	[W·m^−2^·K^−1^]	1.048 × 10^4^ (for cooling)/4.74 × 10^4^ (for warming)
*k_a_*	[s^−1^·K^−1^]	3.933 × 10^7^ (for cooling)/1.287 (for warming)
Crystallisation properties [42,51]		
*T_m_*	[K]	243.5
*Q*	[J·mol^−1^]	4.187 × 10^3^
Other [52]		
*L_h_*	[J·kg^−1^]	334 × 10^3^
$\rho_{h}$	[kg·m^−3^]	1000
$\rho_{w}$	[kg·m^−3^]	2330

**Table 2:** Selected interval results.

Time, *t* [s]	Interval Thermal Conductivity, $\bar{\lambda }$ [W·m^−1^·K^−1^]	Interval Specific Heat Coefficient, $\bar{c}$ × 10^3^ [J·kg^−1^·K^−1^]	Interval Temperature, $\bar{T}$ [°C]	Interval Degree of Crystallisation, $\bar{\chi }$ × 10^−8^
During cooling				
0.0	[0.987; 1.091]	[2.651; 2.930]	[22.000; 22.000]	[0.000; 0.000]
0.1	[2.125; 2.120]	[2.611; 2.612]	[−41.007; −40.677]	[0.3976; 0.3789]
0.2	[2.582; 2.578]	[2.522; 2.523]	[−72.112; −71.873]	[6.108; 6.182]
0.4	[3.195; 3.193]	[2.383; 2.383]	[−121.158; −120.973]	[6.832; 6.932]
0.6	[3.516; 3.515]	[2.295; 2.295]	[−152.115; −152.025]	[6.832; 6.932]
0.8	[3.651; 3.650]	[2.253; 2.253	[−166.894; −166.848]	[6.832; 6.932]
1.0	[3.720; 3.720]	[2.229; 2.229]	[−175.088; −175.040	[6.832; 6.932]
During warming				
0.0	[3.6872; 4.0753]	[2.061; 2.278]	[−196.000; −196.000]	[6.832; 6.932]
0.1	[2.485; 2.496]	[2.542; 2.540]	[−65.211; −65.984]	[1.6 × 10^5^; 1.3 × 10^5^]
0.2	[2.442; 2.443]	[2.551; 2.550]	[−62.254; −62.270]	[5.352 × 10^7^; 5.301 × 10^7^]
0.4	[1.303; 1.310]	[2.750; 2.749]	[7.811; 7.427]	[7.811 × 10^8^; 0.000]
0.6	[0.879; 0.879]	[2.814; 2.814]	[30.397; 30.364]	[0.000; 0.000]
0.8	[0.794; 0.794]	[2.827; 2.827]	[34.752; 34.739]	[0.000; 0.000]
1.0	[0.754; 0.754]	[2.832; 2.832]	[36.765; 36.758]	[0.000; 0.000]

## Data Availability

Not applicable.
